# Supplementary material for: Multi-PGS enhances polygenic prediction by combining 937 polygenic scores
Source: Nat Commun. 2023 Aug 5;14:4702. doi: 10.1038/s41467-023-40330-w (PMC10404269; doi:10.1038/s41467-023-40330-w)
Supplement: Supplementary file 1 — Supplementary Information [file 41467_2023_40330_MOESM1_ESM.pdf]

Supplementary material

Supplementary Methods

PGS library construction 2

1- GWAS summary statistics download 2

2- Parse and format the GWAS summary statistics 2

3- Quality control 4

4- Run LDpred2-auto 4

Supplementary Figures 5-15 **7**

Supplementary Figure 5. AUC iPSYCH linear / non-linear PGS models 7

Supplementary Figure 6. PGS per-quintile odds ratios linear / non-linear PGS models 8

Supplementary Figure 7. ADHD multiPGS lasso weights 9

Supplementary Figure 8. AFF multiPGS lasso weights 10

Supplementary Figure 9. AN multiPGS lasso weights 11

Supplementary Figure 10. ASD multiPGS lasso weights 12

Supplementary Figure 11. BD multiPGS lasso weights 13

Supplementary Figure 12. SCZ multiPGS lasso weights 14

Supplementary Figure 13. Properties of the GWAS summary statistics for top 5 PGS 15

Supplementary Figure 14. PGS per-quintile odds ratios of the different PGS models trained with different data 16

Supplementary Figure 15. ICD10 multiPGS prediction 17

Supplementary references 18

Supplementary Methods

PGS library construction

1- GWAS summary statistics download

A series of resources were used to obtain the GWAS summary statistics for generating PRSs (Supplementary Table 1).

Source	Number of files
GWAS Catalog[1] data freeze 2020-09-09	1376
GWAS ATLAS[2] data freeze v20191115	4756
PGC (exclude iPSYCH samples)	12
Other public sources	62

Supplementary Table 1. Overview of sources for the initial list of GWAS summary statistics.

For the first two data resources, the following filtering was used:

- Total sample > 10,000.
- Number of SNPs in the file > 250,000.
- Training sample of European ancestry. Variables “BROAD ANCESTRAL CATEGORY” for the GWAS Catalog and Population “EUR” or “UKB2 (EUR)” for the GWAS ATLAS.
- For GWAS of the same phenotype, the latest addition to the catalog was selected.
- For GWAS ATLAS, only data from 2014 forward was included.

For the specific PGC GWAS summary statistics where iPSYCH was used in the discovery dataset, we used in-house GWAS results where these samples were excluded from the calculation. **The GWAS were specifically selected to be based on European ancestry individuals, to not be overly redundant and to not contain iPSYCH samples (manually checked).**

For more details, check the script (github script 1-prepare-sumstats.R) that defines the list of GWAS summary statistics from GWAS Catalog and GWAS ATLAS.

Resulting # files: 1,377

For more details, check the script (github script 1-prepare-sumstats.R) that downloaded, read and stored the header and metadata for each GWAS summary statistic file.

2- Parse and format the GWAS summary statistics

We constructed an internal library of GWAS summary statistics header possibilities / column names and filtered out the files with missing essential categories (Supplementary Fig. 1). The essential categories considered were:

chr: chromosome
rsid: rsID number or **pos**: base pair position under hg19
a1: effect allele
a0: no-effect allele
beta: linear / logistic regression effect size
or: odds ratio
beta_se: standard error of effect size
p: p-value

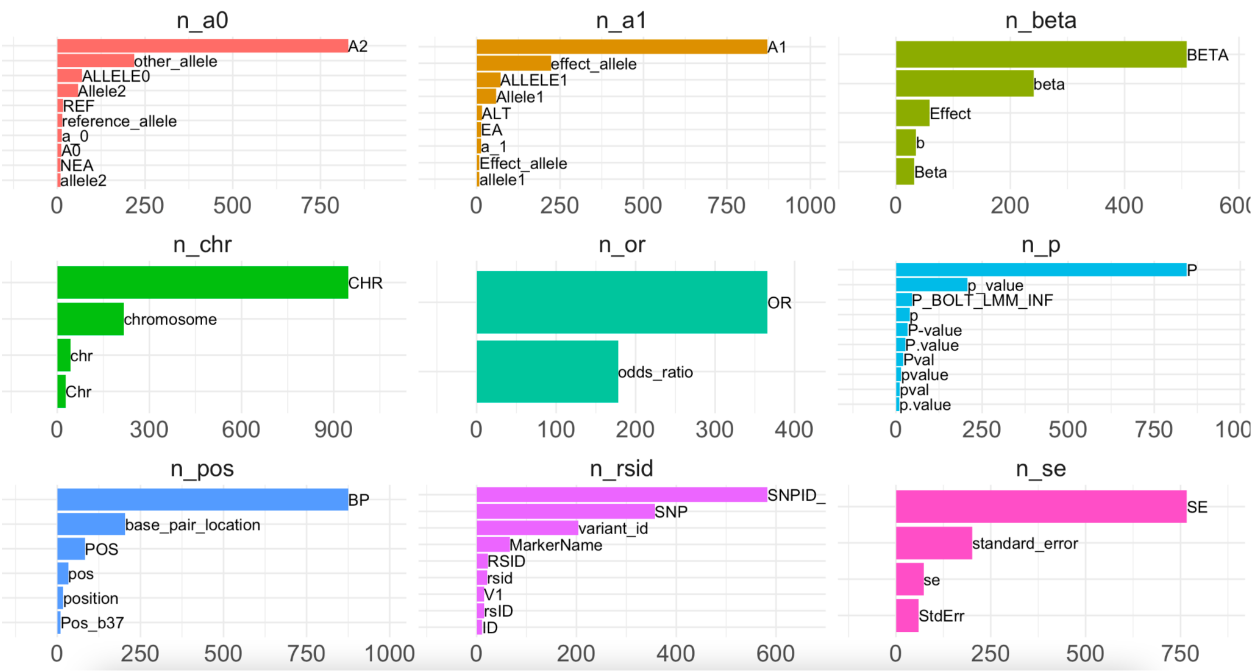

Supplementary Figure 1. Most frequently used column names for the essential categories. Filtered for n>8.

Processing/filters applied:

- Empty beta / or columns or > 30% of the file length is empty → full file discarded.
- rsid column format can't be processed (different string processing tried) → full file discarded.
- Create an effective sample size (required by LDpred2[3], Neff = 4/(1/Ncases + 1/Ncontrol)) column from reading the file (if available), otherwise use metadata.
- Check if the standard error column is from the beta, the log(beta) or the odds ratio and transform if required.
- Check median chi2 statistic (beta/beta_se)^2 for formatted file. If very large → full file discarded.
- Calculate LDSC regression[4] SNP-h2 (prior h2 parameter for LDpred2-auto) and intercept estimates.

Resulting # files: 1,005

For more details, check the script (github script 2-parser.R) that parses and re-formats each GWAS summary statistic file.

3- Quality control

As recommended in the LDpred2 paper[Citation error], filtering SNPs with a large discrepancy in standard deviations between the genotyped/imputed data and the GWAS summary statistics increases the prediction accuracy of PRSs.

Processing/filters applied:

- Filter SNPs to HapMap3 set of variants.
- Filter SNPs to match LD reference set (https://figshare.com/articles/dataset/European_LD_reference_with_blocks_/19213299).
- SNP QC step described in Privé *et al.* 2020[5]
- Make a quality control plot (example on Supplementary Fig. 2).
- Check the number of resulting SNPs. If < 200,000 → full file discarded.

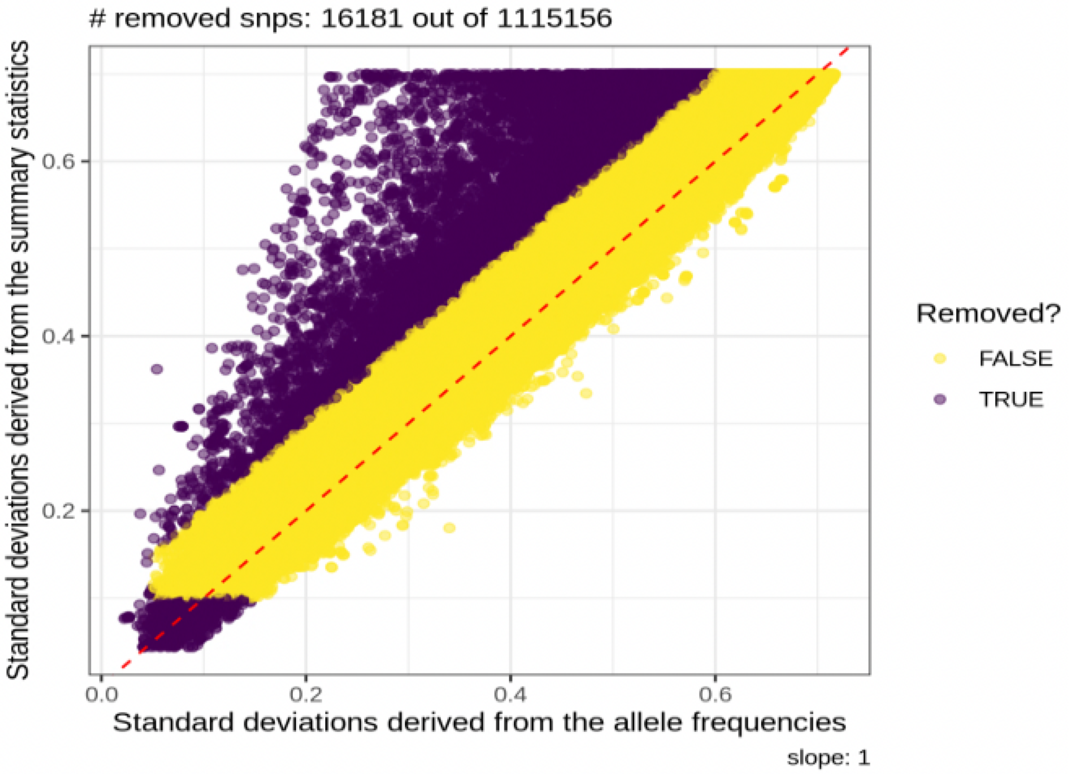

Supplementary Figure 2. Example QC plot. The QC step is described in detail elsewhere[5]**.** The trait shown is for PMID 30643258[6] GWAS summary statistics (Automobile speeding propensity).

Resulting # files: 952

For more details, check the script (github script 3-qc.R) that controls and restricts the number of SNPs in each GWAS summary statistic file.

4- Run LDpred2-auto

Polygenic risk scores were derived using LDpred2-auto, a method within the LDpred2 framework[3] that does not require a validation dataset to fit the hyperparameters (SNP-h2; SNP-based heritability estimate and p; proportion of causal SNPs), but these are fitted as part of the Gibbs sampler instead.

We used the provided European-ancestry independent LD blocks as reference panel[7]. For each GWAS summary statistics file, LDpred2-auto was run with 30 Gibbs sampler chains, 800 burn-in iterations and 400 iterations. The SNP-h2 initial value was set to the LD score regression estimate[8] from the GWAS summary statistics after QC. Each of the chains was initialized with a different value for the proportion of causal variants: in the range [1e-4, 0.9], equally spaced on a log scale. Chains were filtered according to the recommendation in the LDpred2 tutorial, and effect sizes of chains kept were averaged (example convergence plot on Supplementary Fig. 3).

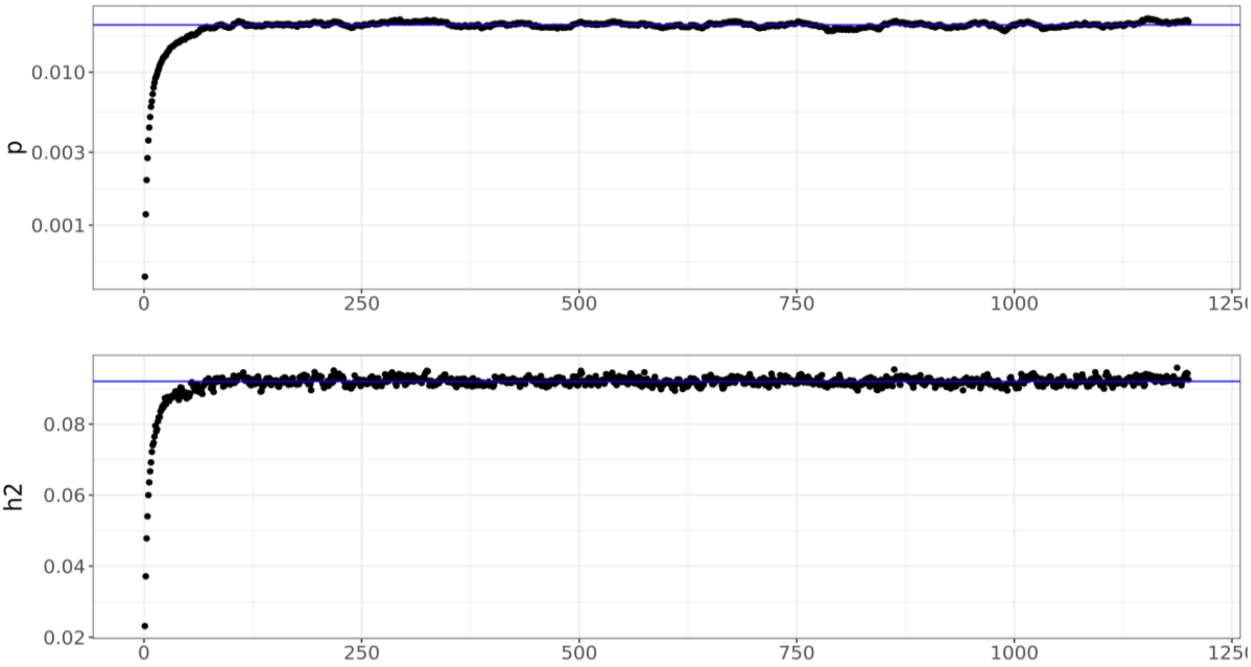

Supplementary Figure 3. Example convergence plot for LDpred2-auto.

Processing/filters applied:

- The LDpred2-auto algorithm did not converge → full file discarded.
- Estimated h2 from LDpred2-auto > 1 → full file discarded.

Resulting # files: 937

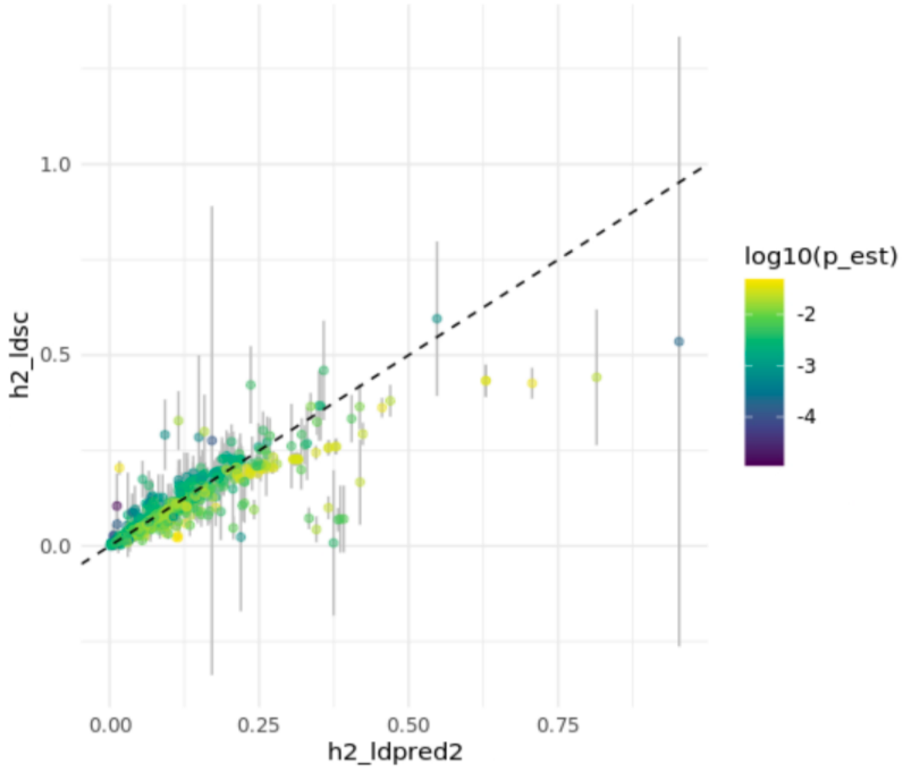

Supplementary Figure 4. Results for the 937 LDpred2-auto polygenic scores in our PGS library. LDSC regression heritability estimates (h2_ldsc, y-axis) +/- se vs. LDpred2-auto heritability estimate (h2_ldpred2, x-axis). The legend indicates the LDpred2-auto estimated proportion of causal variants (p_est).

For more details, check the script (github script 4-run-ldpred2.R) for code on running LDpred2-auto.

Supplementary Figures 5-15

Supplementary Figure 5. AUC iPSYCH linear / non-linear PGS models

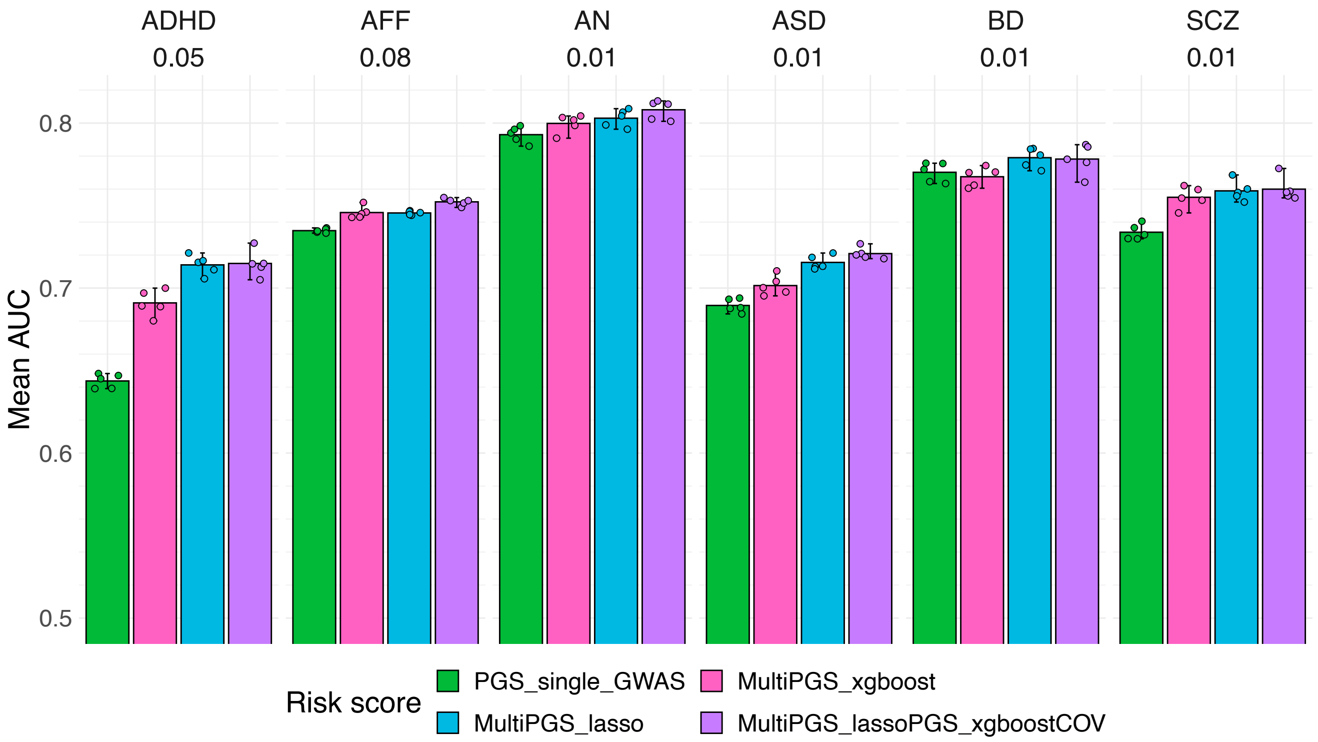

Supplementary Figure 5. Performance of the different risk score models including covariates. Comparison between the per-disorder attention-deficit/hyperactivity disorder (ADHD), affective disorder (AFF), anorexia nervosa (AN), autism spectrum disorder (ASD), bipolar disorder (BD) and schizophrenia (SCZ) single GWAS PGS (specific details on SD2) and the multi-PRS models trained with 937 PGS in terms of AUC. All models included sex, age and first 20 PCs for training the different PGS weights and calculating the risk score on the test set in a 5-fold cross-validation scheme. Confidence intervals were calculated from 10,000 bootstrap samples of the mean adjusted AUC.

Supplementary Figure 6. PGS per-quintile odds ratios linear / non-linear PGS models

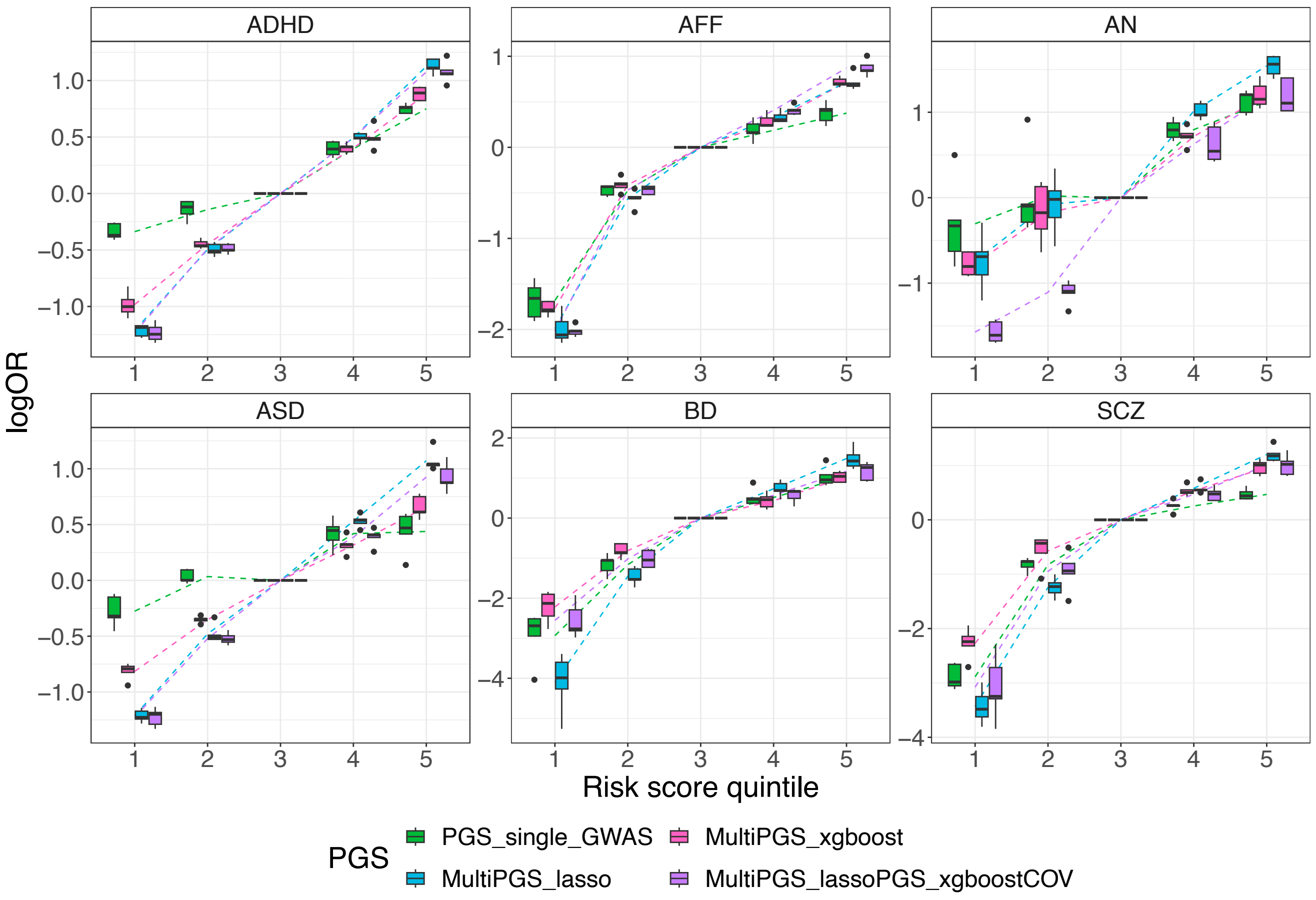

Supplementary Figure 6. Performance of the different risk score models including covariates in terms of risk score quintile log odds ratios (logOR). Comparison between the per-disorder attention-deficit/hyperactivity disorder (ADHD), affective disorder (AFF), anorexia nervosa (AN), autism spectrum disorder (ASD), bipolar disorder (BD) and schizophrenia (SCZ) single GWAS PGS (specific details on SD2) and the multi-PRS models trained with 937 PGS in terms of the logOR of the risk score quintiles compared to the middle quintile. All models included sex, age and first 20 PCs for training the different PGS weights and calculating the risk score on the test set in a 5-fold cross-validation scheme. Confidence intervals were calculated from 10,000 bootstrap samples of the mean logOR.

Supplementary Figure 7. ADHD multiPGS lasso weights

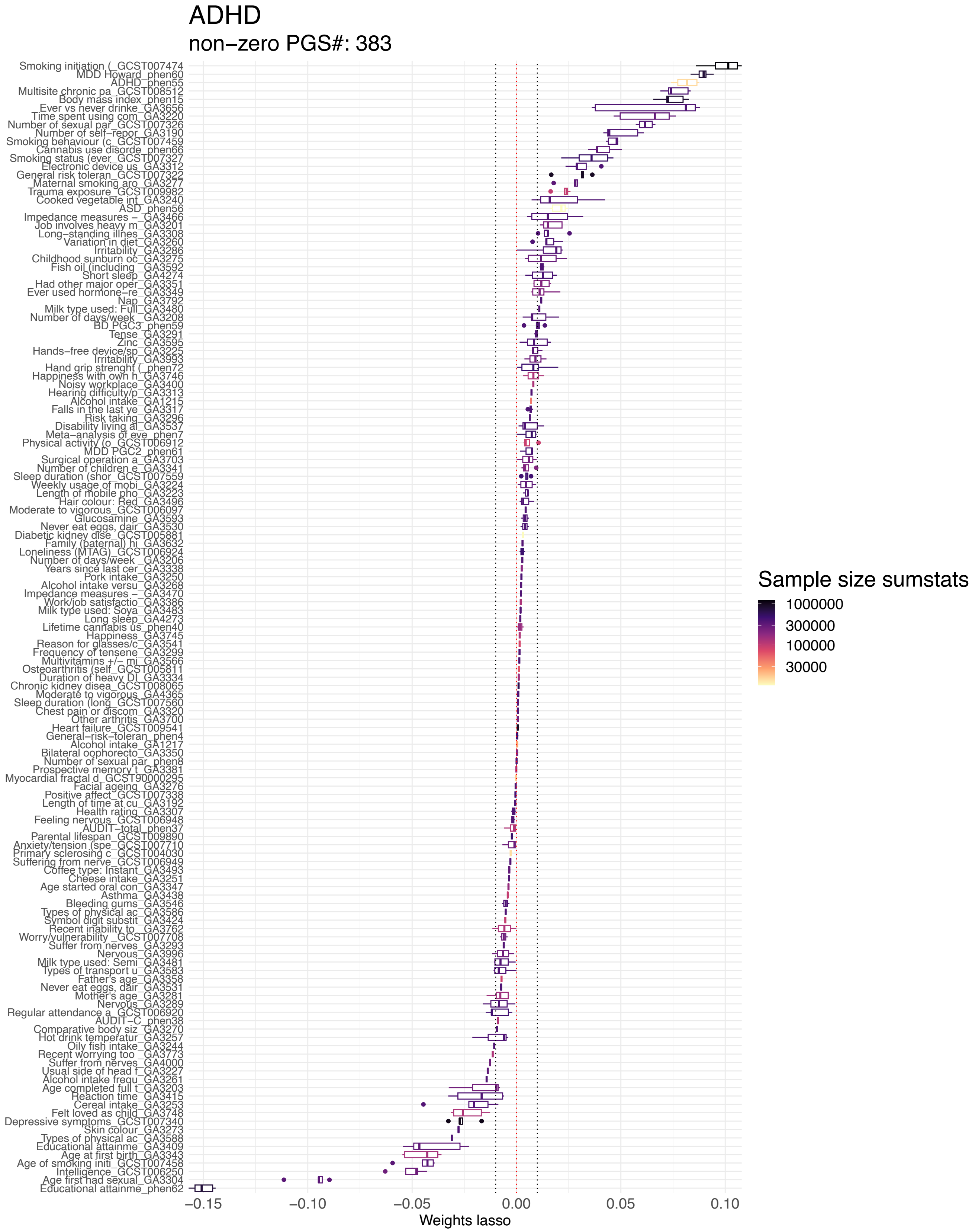

Supplementary Figure 7. ADHD multiPGS lasso weights. Mean lasso weight (x-axis) for the 5 cross-validation subsets of the lasso multiPGS. Each PGS with a non-zero lasso weight is represented by the y-axis with GWAS outcome name and the unique GWAS identifier (SD1). The color indicates the sample size of the external GWAS summary statistics for each PGS. The number of variables in the y-axis is available in the subtitle.

Supplementary Figure 8. AFF multiPGS lasso weights

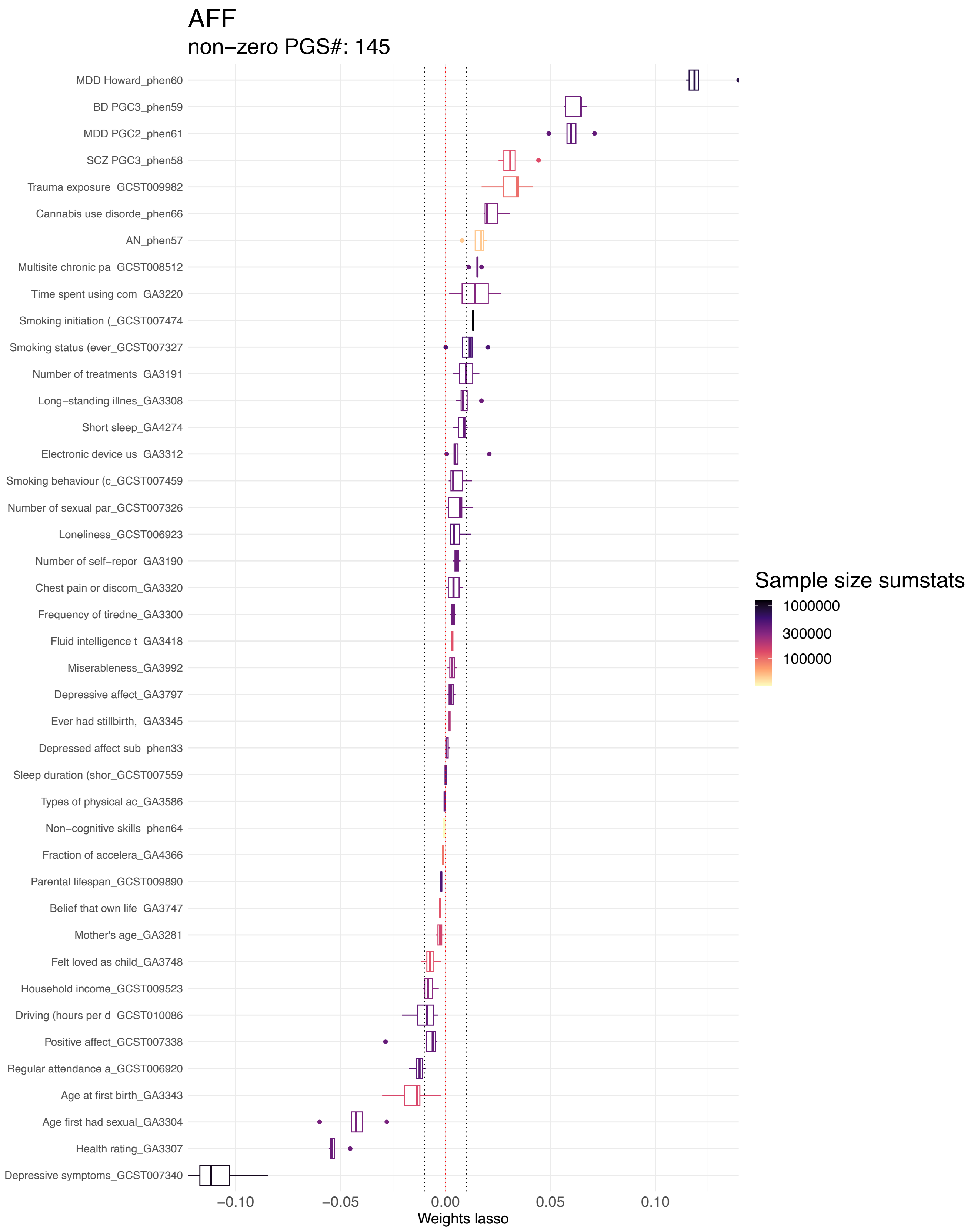

Supplementary Figure 8. AFF multiPGS lasso weights. Mean lasso weight (x-axis) for the 5 cross-validation subsets of the lasso multiPGS. Each PGS with a non-zero lasso weight is represented by the y-axis with GWAS outcome name and the unique GWAS identifier (SD1). The color indicates the sample size of the external GWAS summary statistics for each PGS. The number of variables in the y-axis is available in the subtitle.

Supplementary Figure 9. AN multiPGS lasso weights

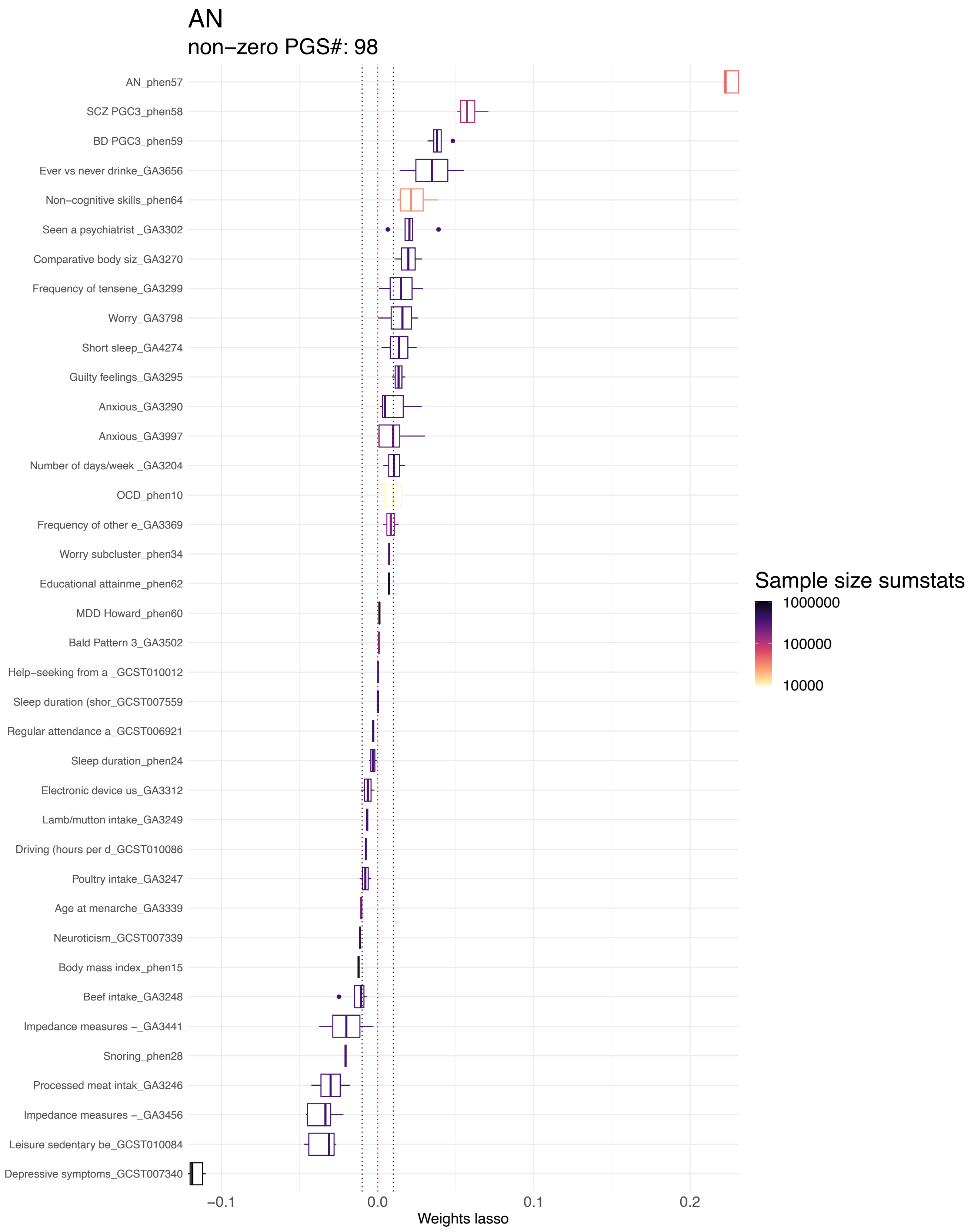

Supplementary Figure 9. AN multiPGS lasso weights. Mean lasso weight (x-axis) for the 5 cross-validation subsets of the lasso multiPGS. Each PGS with a non-zero lasso weight is represented by the y-axis with GWAS outcome name and the unique GWAS identifier (SD1). The color indicates the sample size of the external GWAS summary statistics for each PGS. The number of variables in the y-axis is available in the subtitle.

Supplementary Figure 10. ASD multiPGS lasso weights

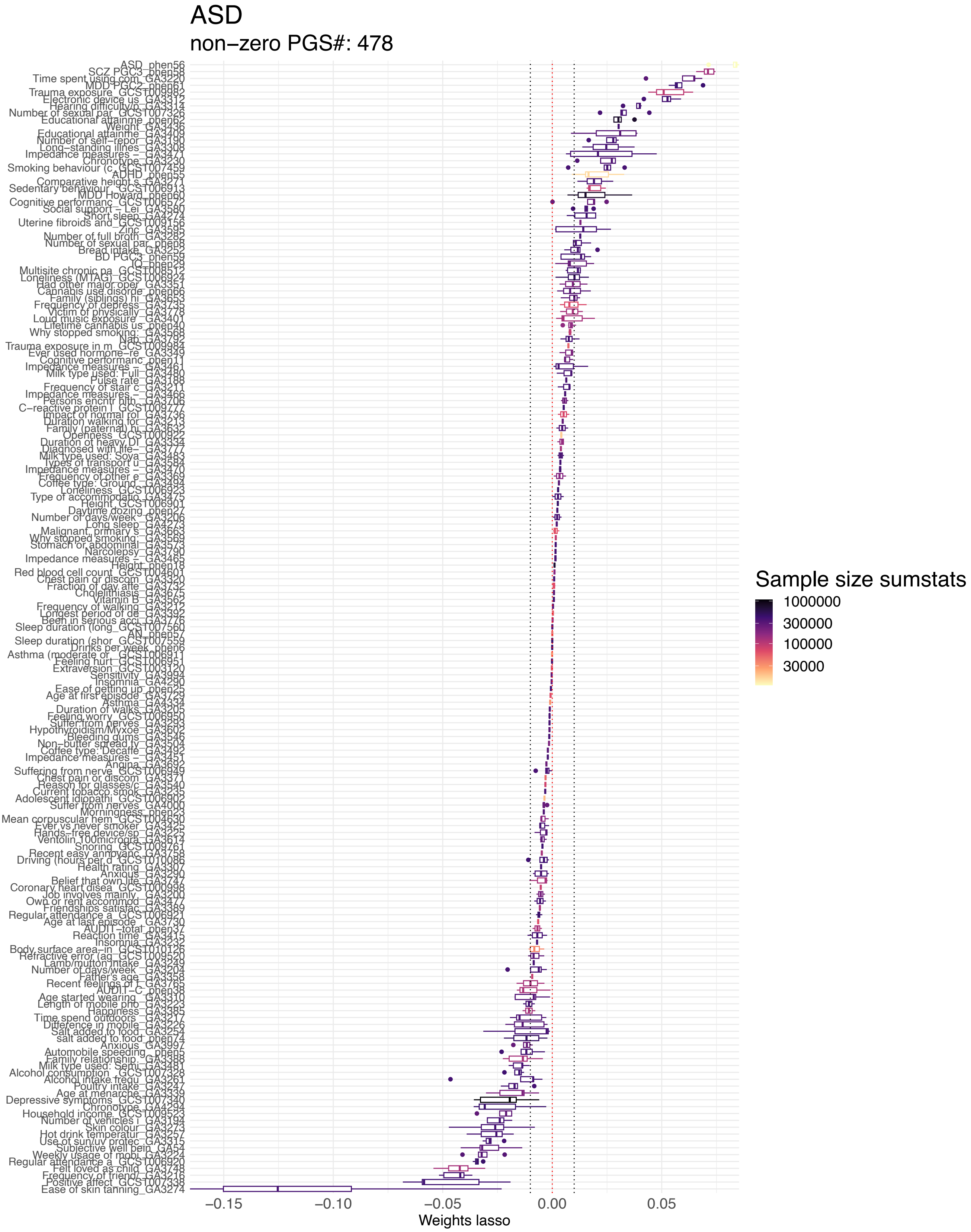

Supplementary Figure 10. ASD multiPGS lasso weights. Mean lasso weight (x-axis) for the 5 cross-validation subsets of the lasso multiPGS. Each PGS with a non-zero lasso weight is represented by the y-axis with GWAS outcome name and the unique GWAS identifier (SD1). The color indicates the sample size of the external GWAS summary statistics for each PGS. The number of variables in the y-axis is available in the subtitle.

Supplementary Figure 11. BD multiPGS lasso weights

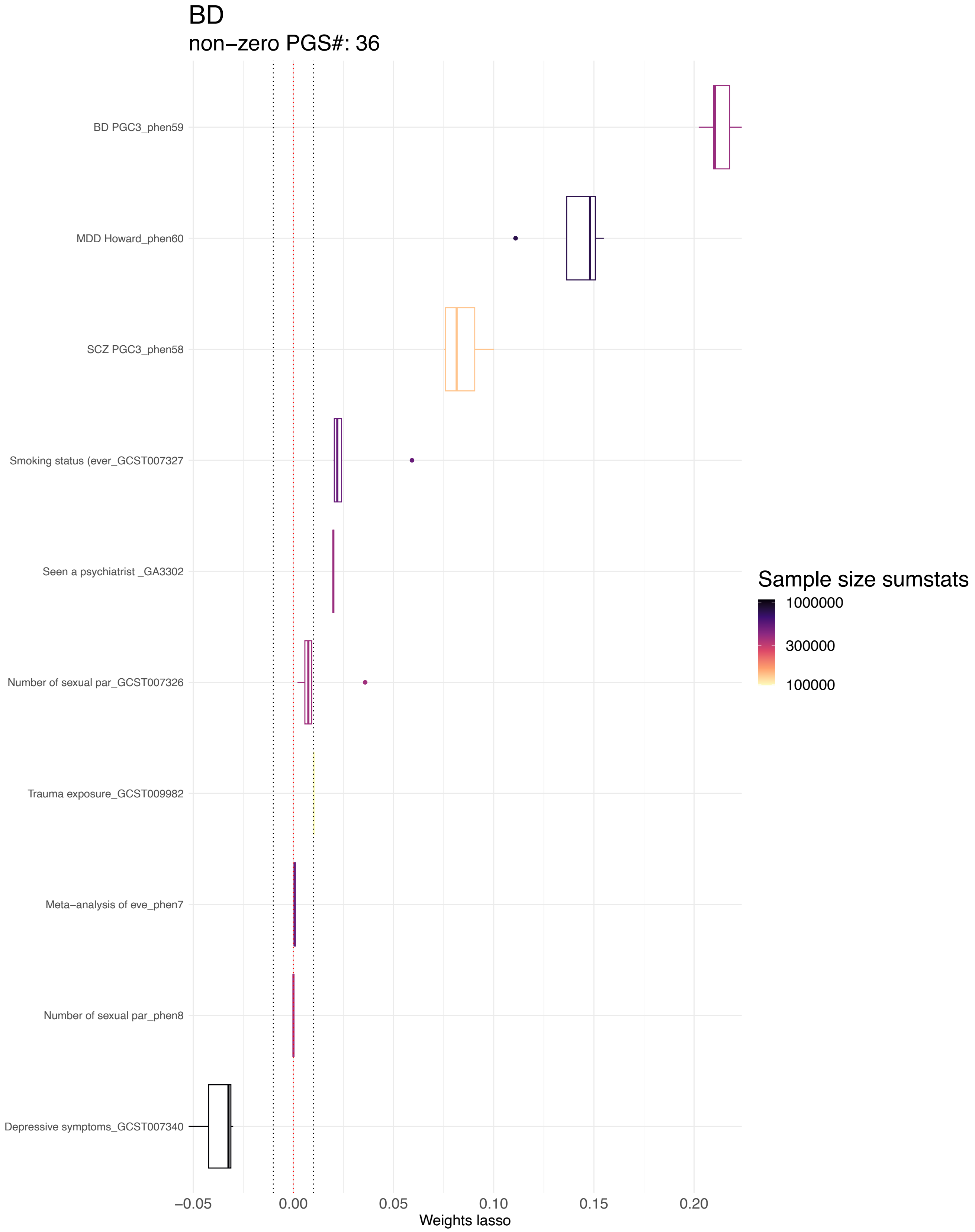

Supplementary Figure 11. BD multiPGS lasso weights. Mean lasso weight (x-axis) for the 5 cross-validation subsets of the lasso multiPGS. Each PGS with a non-zero lasso weight is represented by the y-axis with GWAS outcome name and the unique GWAS identifier (SD1). The color indicates the sample size of the external GWAS summary statistics for each PGS. The number of variables in the y-axis is available in the subtitle.

Supplementary Figure 12. SCZ multiPGS lasso weights

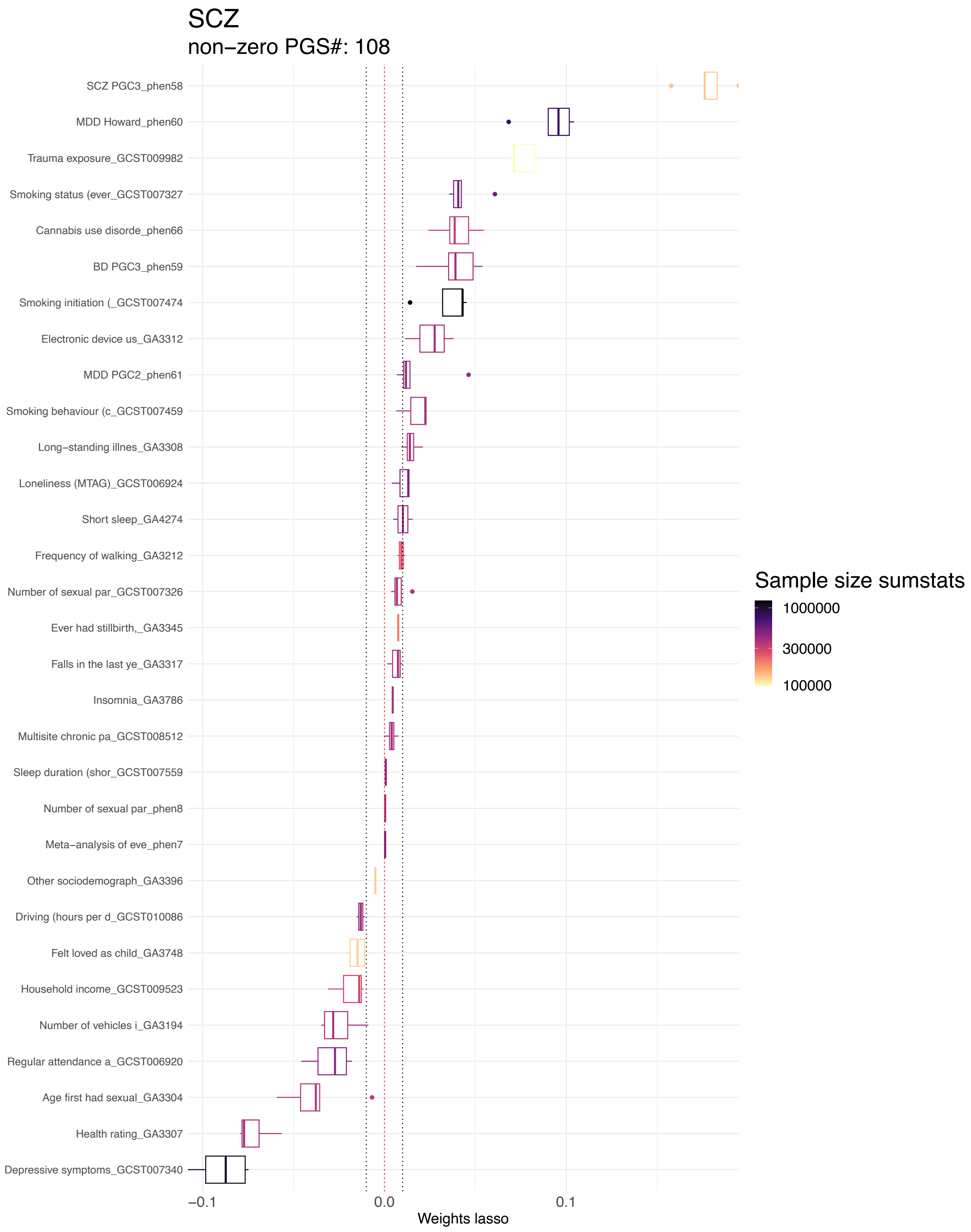

Supplementary Figure 12. SCZ multiPGS lasso weights. Mean lasso weight (x-axis) for the 5 cross-validation subsets of the lasso multiPGS. Each PGS with a non-zero lasso weight is represented by the y-axis with GWAS outcome name and the unique GWAS identifier (SD1). The color indicates the sample size of the external GWAS summary statistics for each PGS. The number of variables in the y-axis is available in the subtitle.

Supplementary Figure 13. Properties of the GWAS summary statistics for top 5 PGS

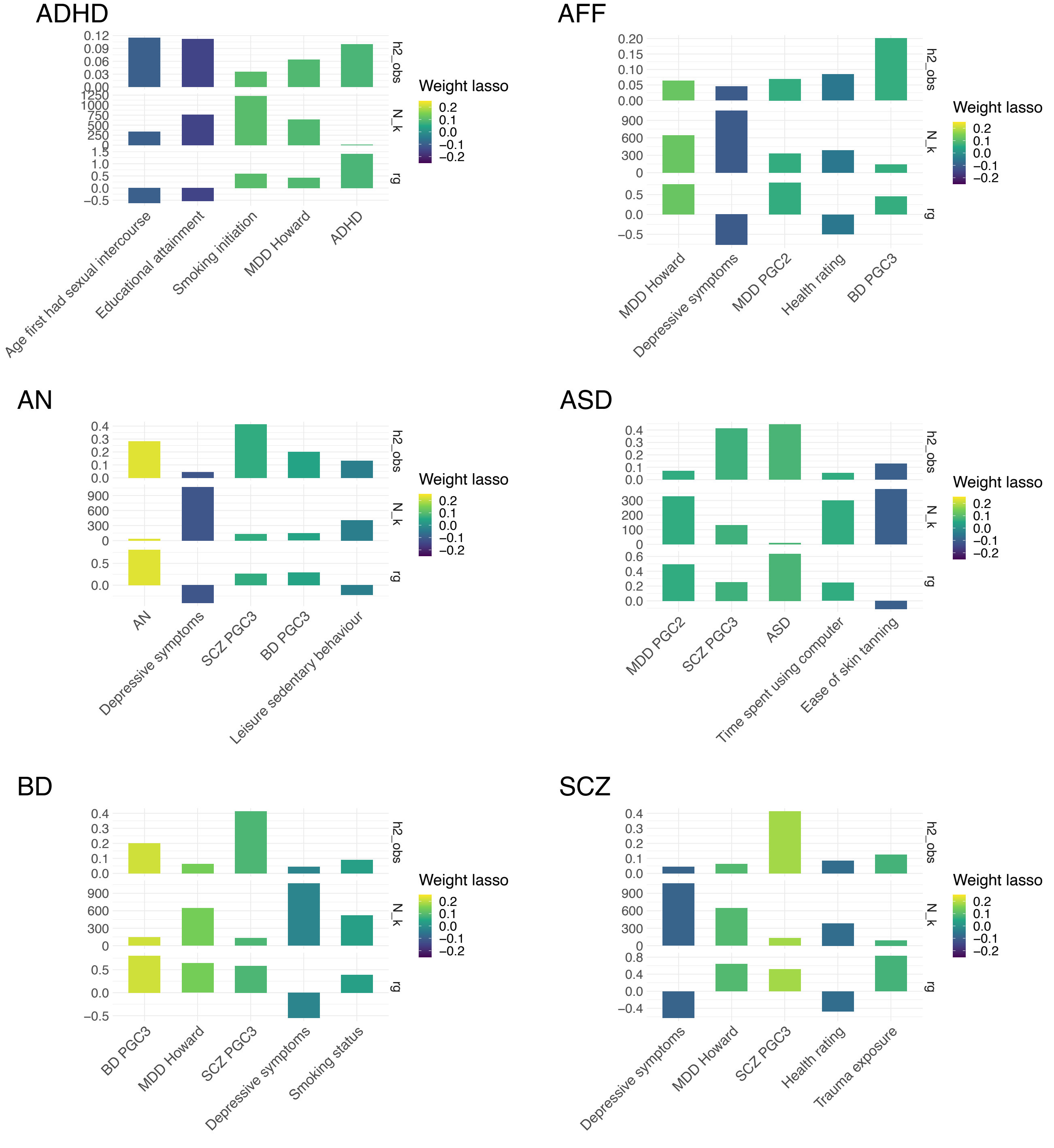

Supplementary Figure 13. Properties of the GWAS summary statistics for top 5 PGS. Top 5 PGS based on the lasso weight (x-axis) for attention-deficit/hyperactivity disorder (ADHD), affective disorder (AFF), anorexia nervosa (AN), autism spectrum disorder (ASD), bipolar disorder (BD) and schizophrenia (SCZ), with SNP heritability in the observed scale (h2_obs), sample size in thousands (N_k) and genetic correlation with the predicted trait (rg) (from top to bottom in each panel).

Supplementary Figure 14. PGS per-quintile odds ratios of the different PGS models trained with different data

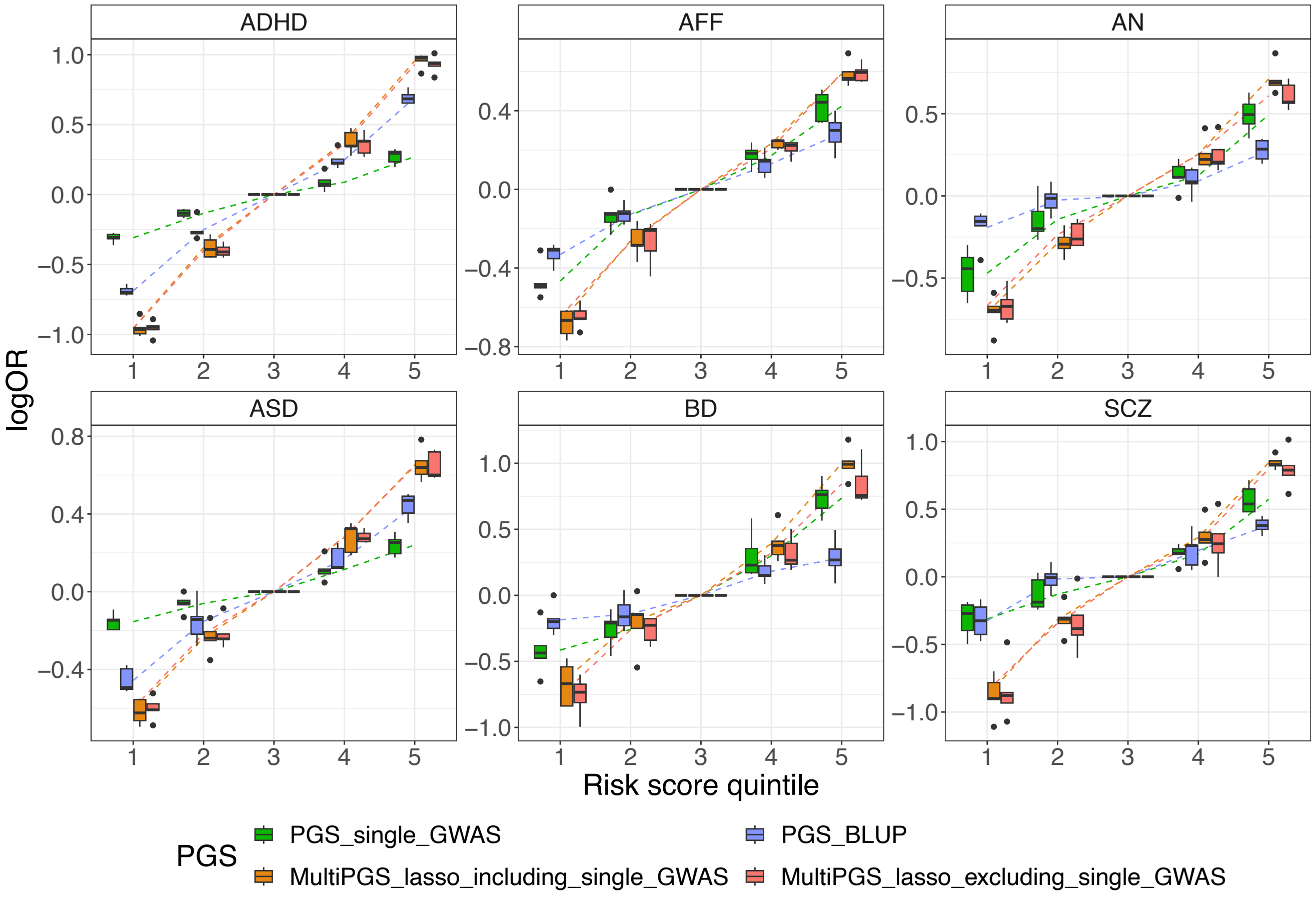

Supplementary Figure 14. Performance of the different PGS models trained with different data. Comparison between the per-disorder attention-deficit/hyperactivity disorder (ADHD), affective disorder (AFF), anorexia nervosa (AN), autism spectrum disorder (ASD), bipolar disorder (BD) and schizophrenia (SCZ) single GWAS PGS (PGS_single_GWAS) (Details on SD2), the per-disorder BLUP PGS and the multi-PGS models in terms of the logOR of the risk score quintiles compared to the middle quintile. The multiPGS_lasso_excluding_single_GWAS represents the PGS where the specific single GWAS PGS was removed from the set of 937 PGSAll models included sex, age and first 20 PCs for training the different PGS weights and calculating the risk score on the test set in a 5-fold cross-validation scheme. Confidence intervals were calculated from 10,000 bootstrap samples of the mean logOR.

Supplementary Figure 15. ICD10 multiPGS prediction

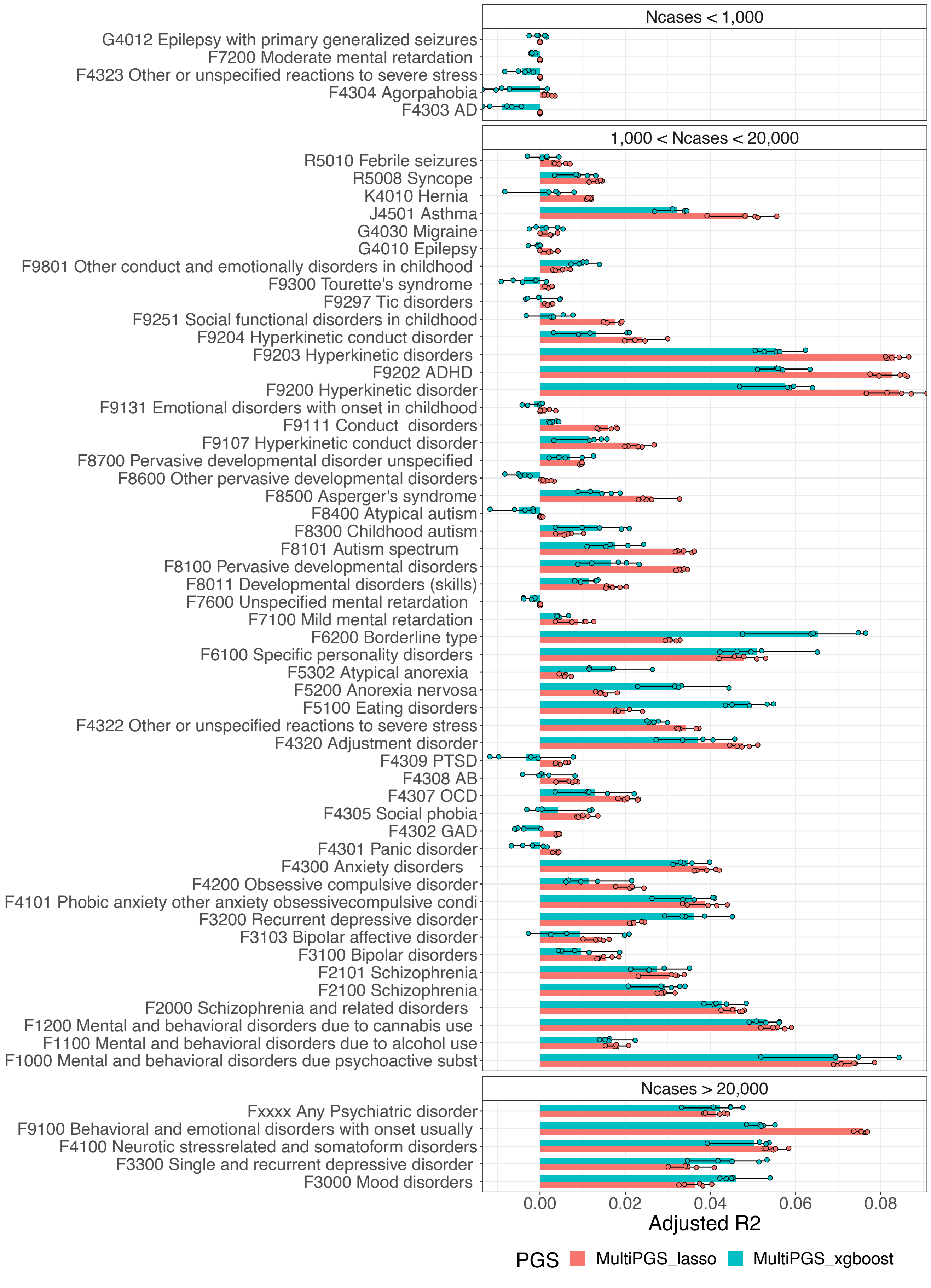

Supplementary Figure 15. Performance of the multi-PGS on register-based outcomes. Comparison between the per-disorder multi-PRS models trained with 937 PGS adjusted R2. All models included sex, age and first 20 PCs for training the different PGS weights and calculating the risk score on the test set in a 5-fold cross-validation scheme. Confidence intervals were calculated from 10,000 bootstrap samples of the mean adjusted R2, where the adjusted R2 was the variance explained by the full model after accounting for the variance explained by a logistic regression covariates-only model as R2_adjusted = (R2_full - R2_cov) / (1 - R2_cov).

Supplementary references

1. Buniello, A. *et al.* The NHGRI-EBI GWAS Catalog of published genome-wide association studies, targeted arrays and summary statistics 2019. *Nucleic Acids Res.* **47**, D1005–D1012 (2019).
2. Watanabe, K. *et al.* A global overview of pleiotropy and genetic architecture in complex traits. *Nat. Genet.* **51**, 1339–1348 (2019).
3. Privé, F., Arbel, J. & Vilhjálmsson, B. J. LDpred2: better, faster, stronger. *Bioinformatics* (2020) doi:10.1093/bioinformatics/btaa1029.
4. Bulik-Sullivan, B. K. *et al.* LD Score regression distinguishes confounding from polygenicity in genome-wide association studies. *Nature Genetics* vol. 47 291–295 Preprint at https://doi.org/10.1038/ng.3211 (2015).
5. Privé, F., Arbel, J., Aschard, H. & Vilhjálmsson, B. J. Identifying and correcting for misspecifications in GWAS summary statistics and polygenic scores. *HGG Adv* **3**, 100136 (2022).
6. Karlsson Linnér, R. *et al.* Genome-wide association analyses of risk tolerance and risky behaviors in over 1 million individuals identify hundreds of loci and shared genetic influences. *Nat. Genet.* **51**, 245–257 (2019).
7. Privé, F., Arbel, J., Aschard, H. & Vilhjálmsson, B. J. Identifying and correcting for misspecifications in GWAS summary statistics and polygenic scores. *bioRxiv* 2021.03.29.437510 (2022) doi:10.1101/2021.03.29.437510.
8. Bulik-Sullivan, B. K. *et al.* LD Score regression distinguishes confounding from polygenicity in genome-wide association studies. *Nat. Genet.* **47**, 291–295 (2015).
