# Supplementary material for: Multi-PGS enhances polygenic prediction by combining 937 polygenic scores
Source: Nat Commun. 2023 Aug 5;14:4702. doi: 10.1038/s41467-023-40330-w (PMC10404269; doi:10.1038/s41467-023-40330-w)
Supplement: Supplementary file 3 — Description of Additional Supplementary Files [file 41467_2023_40330_MOESM3_ESM.pdf]

Description of Additional Supplementary Files

File Name: Supplementary Data 1
Description: Meta-data and results PGS library. Information on the 937 GWAS summary statistics with LDpred2-auto PGS that were the input to the multiPGS models. GWAS_trait: reported outcome of GWAS, id: unique identifier of GWAS, GWAS_pubmedID: PubMed ID of GWAS, source: file downloaded from this resource, M_or: original number of variants in GWAS summary statistics file, M_m: number of variants that matches the iPSYCH HM3 subset, M_ldpred: number of variants that matches the LDpred2 provided LD reference for European ancestry and passes QC (described in Supplementary Text), k: number of Gibbs sampler chains that passes the LDpred2 recommended filter (out of 30), h2_est: LDpred2-auto estimated SNP-heritability parameter, p_est: LDpred2-auto estimated proportion of causal variants parameter.

File Name: Supplementary Data 2
Description: Summary of main iPSYCH outcomes and compared PGS.
Information on the 6 psychiatric disorders: attention-deficit/hyperactivity disorder (ADHD), affective disorder (AFF), anorexia nervosa (AN), autism spectrum disorder (ASD), bipolar disorder (BD) and schizophrenia (SCZ) in the main analysis. Tag: outcome label, p: ICD10 category, cv: 5-fold cross-validation subset, prev_pop: population prevalence used for liability-scale transformation, prev_gwas: individual-level data case-control ratio used for liability-scale transformation, n_cases_train: number of cases in training subset, n_control_train, number of controls in training subset, n_cases_test: number of cases in testing subset, n_control_test: number of controls in testing subset, comp: label for the GWAS summary statistics used to compute the PGS to compare against, GWAS_trait, compared outcome, GWAS_pubmedID: PubMed ID of GWAS, id: unique identifier of GWAS for meta-data PGS library, n_cases_GWAS: number of cases in GWAS, n_control_GWAS: number of controls in GWAS.

File Name: Supplementary Data 3
Description: List of all phenotypes and ICD10 codes. First cross-validation subset information for all used phenotypes and ICD10 codes in the analyses, including information on the sample overlap with the iPSYCH cohort and the main diagnosis. p: ICD10 category, diagnosis: description of the ICD10 category, cases: number of cases in first cross-validation subset, scz, adhd, asd, aff, bip, cohort: proportion of cases that overlap with the different groups.

File Name: Supplementary Data 4
Description: Information on the 15 traits selected for Figure 5. For ADHD/ASD, ASD was coded as 0 (controls) and ADHD was coded as 1 (cases). For BD/MDD, MDD was coded as 0 (controls) and BD was coded as 1 (cases). Tag: outcome label, p: ICD10 category, cv: 5-fold cross-validation subset, n_cases_train: number of cases in training subset, n_control_train, number of controls in training subset, n_cases_test: number of cases in testing subset, n_control_test: number of controls in testing subset, phen_comp: phenotype ID from the top-weighted lasso PGS in the model , GWAS_trait: label for the phen_comp phenotype ID, PubMed ID: PubMed ID of GWAS,, N_GWAS: sample size for continuous phenotypes, n_cases_GWAS: number of cases in GWAS, n_control_GWAS: number of

controls in GWAS, prev_gwas: individual-level data case-control ratio used for liability-scale transformation.
