# Supplementary material for: Multi-PGS enhances polygenic prediction by combining 937 polygenic scores
Source: Nat Commun. 2023 Aug 5;14:4702. doi: 10.1038/s41467-023-40330-w (PMC10404269; doi:10.1038/s41467-023-40330-w)
Supplement: Supplementary file 8 — Reporting Summary [file 41467_2023_40330_MOESM8_ESM.pdf]

nature portfolio

Corresponding author(s): Clara Albiñana

Last updated by author(s): Jun 29, 2023

Reporting Summary

Nature Portfolio wishes to improve the reproducibility of the work that we publish. This form provides structure for consistency and transparency in reporting. For further information on Nature Portfolio policies, see our Editorial Policies and the Editorial Policy Checklist.

Statistics

For all statistical analyses, confirm that the following items are present in the figure legend, table legend, main text, or Methods section.

n/a	Confirmed	
☐	☒	The exact sample size (*n*) for each experimental group/condition, given as a discrete number and unit of measurement
☐	☒	A statement on whether measurements were taken from distinct samples or whether the same sample was measured repeatedly
☐	☒	The statistical test(s) used AND whether they are one- or two-sided<br>*Only common tests should be described solely by name; describe more complex techniques in the Methods section.*
☐	☒	A description of all covariates tested
☐	☒	A description of any assumptions or corrections, such as tests of normality and adjustment for multiple comparisons
☐	☒	A full description of the statistical parameters including central tendency (e.g. means) or other basic estimates (e.g. regression coefficient) AND variation (e.g. standard deviation) or associated estimates of uncertainty (e.g. confidence intervals)
☐	☒	For null hypothesis testing, the test statistic (e.g. F, t, r) with confidence intervals, effect sizes, degrees of freedom and P value noted<br>*Give P values as exact values whenever suitable.*
☒	☐	For Bayesian analysis, information on the choice of priors and Markov chain Monte Carlo settings
☒	☐	For hierarchical and complex designs, identification of the appropriate level for tests and full reporting of outcomes
☐	☒	Estimates of effect sizes (e.g. Cohen's *d*, Pearson's *r*), indicating how they were calculated

Our web collection on statistics for biologists contains articles on many of the points above.

Software and code

Policy information about availability of computer code

Data collection	This is fully described in the Online methods and associated supplementary text.<br>Genotyping of the iPSYCH2012 sample was performed at the Broad Institute of Harvard and MIT (Cambridge, MA, USA) with PsychChip arrays from Illumina according to the manufacturer's instructions. Genotyping of the iPSYCH2015i sample was pefromed at the Staten Serum Institute (SSI, Copenhagen, Denmark) using the Global Screening Array v2 with a Multi disease drop in (Illumina, San Diego, California) following the manufacturer's instructions.<br>We utilized the list of GWAS Catalog (https://www.ebi.ac.uk/gwas/) summary statistics downloaded on 09/09/2020, GWAS summary statistics from the PGC (https://www.med.unc.edu/pgc/download-results/) and GWAS Atlas UKB2 data freeze v20191115 (https://atlas.ctglab.nl/).
Data analysis	This is fully described in the Online methods and associated supplementary text. All code used for analysis of this manuscript is available at https://github.com/ClaraAlbi/paper_multiPGS<br><br>Preimputation quality control was performed using Ricopili pipeline (https://sites.google.com/a/broadinstitute.org/ricopili/)<br>Principal components were derived using the R package bigsnpr (function snp_pcadapt)<br>Polygenic scores were derived with LDpred2-auto using the R package bigsnpr (function snp_ldpred2_auto)<br>BOLT-LMM (https://data.broadinstitute.org/alkesgroup/bolt-lmm),<br>XGBoost (https://xgboost.readthedocs.io/en/latest/), SMTpred<br>(https://github.com/uqrmaie1/smtpred)

For manuscripts utilizing custom algorithms or software that are central to the research but not yet described in published literature, software must be made available to editors and reviewers. We strongly encourage code deposition in a community repository (e.g. GitHub). See the Nature Portfolio guidelines for submitting code & software for further information.

Data

Policy information about availability of data

All manuscripts must include a data availability statement. This statement should provide the following information, where applicable:

- Accession codes, unique identifiers, or web links for publicly available datasets
- A description of any restrictions on data availability
- For clinical datasets or third party data, please ensure that the statement adheres to our policy

The multi-PGS lasso weights generated in this study have been deposited in the figshare database (https://doi.org/10.6084/m9.figshare.23597019.v1). The iPSYCH and Danish ANGI data are available under restricted access as the data are protected by Danish legislation, access can be obtained after approval by the iPSYCH Data Access Committee and can only be accessed on the secured Danish server GenomeDK (https://genome.au.dk). For data access and correspondence, please contact C.A. (clara@au.dk) or B.J.V. (bjv.ncrr@au.dk). The PGS library metadata generated in this study is provided in the Supplementary Information/Source Data file. The GWAS summary statistics data used in this study are available in the GWAS Catalog database (https://www.ebi.ac.uk/gwas/, downloaded on 09/09/2020), GWAS Atlas UKB2 data freeze v20191115 (https://atlas.ctglab.nl/) and PGC downloads (https://www.med.unc.edu/pgc/download-results/).

Research involving human participants, their data, or biological material

Policy information about studies with human participants or human data. See also policy information about sex, gender (identity/presentation), and sexual orientation and race, ethnicity and racism.

Reporting on sex and gender	The research findings reported apply to both sexes, and sex is not mentioned in the title. Within the iPSYCH sample, sex was assigned based on information from the National registers and subsequently checked using genetic markers on the chromosome.
Reporting on race, ethnicity, or other socially relevant groupings	We report results for individuals tagged as having "European" ancestry. This group was identified by defining a homogeneous group of individuals using the first 20 principal components, with a radius around the center of the sample. This European group represents >70% of the total sample.
Population characteristics	The iPSYCH 2015 sample is a population case-cohort sample that comprises individuals born in Denmark between May 1, 1981 and December 31st, 2008, who were alive and resided in Denmark on their first birthday.<br>The iPSYCH sample was processed in two matches (genotyping, qc and imputation done separately). All analyses were adjusted for batch, sex, birth year and the first 20 principal components to control for population stratification.<br>The full sample consists of 52% females, 60% on batch 1 (iPSYCH2012) and the mean birth year is 1993 (Q1-1988 Q3-1999)
Recruitment	This sample is population based, with individuals identified from the National Registried and the blood samples were pulled from the Danish Neonatal Screening Biobank.
Ethics oversight	The study was approved by the local scientific ethics committees and IRBs. The iPSYCH study was approved by the Scientific Ethics Committee in the Central Denmark Region (Case no 1-10-72-287-12) and the Danish Data Protection Agency. In accordance with Danish legislation, the Danish Scientific Ethics commitee has, for this study, waived the need for specific informed consent in biomedical research based on existing biobanks.

Note that full information on the approval of the study protocol must also be provided in the manuscript.

Field-specific reporting

Please select the one below that is the best fit for your research. If you are not sure, read the appropriate sections before making your selection.

☒ Life sciences ☐ Behavioural & social sciences ☐ Ecological, evolutionary & environmental sciences

For a reference copy of the document with all sections, see nature.com/documents/nr-reporting-summary-flat.pdf

Life sciences study design

All studies must disclose on these points even when the disclosure is negative.

Sample size	No sample size calculation was made. Previous studies of psychiatric disorders have demonstrated that high numbers of cases and controls yield enough power to detect common risk variants with low effect sizes.
Data exclusions	Analyses were restricted to individuals of European ancestry and unrelated individuals with each pair being >3rd degree relatedness.
Replication	We applied a 5-fold cross validation technique to our sample to provide out-of-sample accuracies, which is a pseudo-replication tecnique.
Randomization	It is an observational study, comparing cases and controls based on diagnosis obtained from registers and a random population-based sample, no randomization was performed as such.
Blinding	In iPSYCH, diagnosis are drawn from registers. These are administrative data bases populated by data from the clinicians long before the

Blinding	current study. The blood samples are pulled from a biobank. Hence, the study participants and diagnosing clinicians are blinded with respect to the study. The iPSYCH dataset was generated with an overall goal of investigating the genetic and environmental effects of psychiatric disorders.

Reporting for specific materials, systems and methods

We require information from authors about some types of materials, experimental systems and methods used in many studies. Here, indicate whether each material, system or method listed is relevant to your study. If you are not sure if a list item applies to your research, read the appropriate section before selecting a response.

Materials & experimental systems

n/a	Involved in the study
☒	☐ Antibodies
☒	☐ Eukaryotic cell lines
☒	☐ Palaeontology and archaeology
☒	☐ Animals and other organisms
☒	☐ Clinical data
☒	☐ Dual use research of concern
☒	☐ Plants

Methods

n/a	Involved in the study
☒	☐ ChIP-seq
☒	☐ Flow cytometry
☒	☐ MRI-based neuroimaging
